# Challenging popular tools for the annotation of genetic variations with a real case, pathogenic mutations of lysosomal alpha-galactosidase

**DOI:** 10.1186/s12859-018-2416-7

**Published:** 2018-11-30

**Authors:** Chiara Cimmaruta, Valentina Citro, Giuseppina Andreotti, Ludovica Liguori, Maria Vittoria Cubellis, Bruno Hay Mele

**Affiliations:** 1 Dipartimento di Biologia, Complesso di Monte Sant’Angelo, Università Federico II, VIA Cinthia, 80126 Napoli, Italy; 2 Istituto di Chimica Biomolecolare, Consiglio Nazionale delle Ricerche, Pozzuoli, Italy; 3 Dipartimento di Scienze e Tecnologie Ambientali, Biologiche e Farmaceutiche, Università della Campania “Luigi Vanvitelli”, Caserta, Italy; 4 Dipartimento di Agraria, Università Federico II, Napoli, Italy

**Keywords:** Rare disease, Clinical informatics, Variant analysis, Bioinformatics, Fabry disease

## Abstract

**Background:**

Severity gradation of missense mutations is a big challenge for exome annotation. Predictors of deleteriousness that are most frequently used to filter variants found by next generation sequencing, produce qualitative predictions, but also numerical scores. It has never been tested if these scores correlate with disease severity.

**Results:**

wANNOVAR, a popular tool that can generate several different types of deleteriousness-prediction scores, was tested on Fabry disease. This pathology, which is caused by a deficit of lysosomal alpha-galactosidase, has a very large genotypic and phenotypic spectrum and offers the possibility of associating a quantitative measure of the damage caused by mutations to the functioning of the enzyme in the cells. Some predictors, and in particular VEST3 and PolyPhen2 provide scores that correlate with the severity of lysosomal alpha-galactosidase mutations in a statistically significant way.

**Conclusions:**

Sorting disease mutations by severity is possible and offers advantages over binary classification. Dataset for testing and training in silico predictors can be obtained by transient transfection and evaluation of residual activity of mutants in cell extracts. This approach consents to quantitative data for severe, mild and non pathological variants.

**Electronic supplementary material:**

The online version of this article (10.1186/s12859-018-2416-7) contains supplementary material, which is available to authorized users.

## Background

Exome sequencing has become very popular for the diagnosis of genetic diseases.This is certainly due to high-throughput platforms that have greatly reduced the costs of sequences and to the tools for the analysis of data that are freely available to researchers. Pipelines for the processing [1] and the annotation of data have been proposed with the intent of “democratizing the ability to compile information on large amounts of genetic variations in individual laboratories” [2]. A critical step in the annotation process is represented by the evaluation of missense mutations. A popular annotation tool, wANNOWAR [3], can generate several different types of deleteriousness-prediction scores running SIFT [4], LRT [5], MutationAssessor [6], FATHMM [7], PROVEAN [8], VEST3 [9] metaSVM [10], metaLR [10], M-CAP [11], PolyPhen-2 [12], MutationTaster [13], CADD [14], DANN [15], fathmm-MKL coding [16], GenoCanyon [17], GERP++, [18, 19], phyloP7way vertebrate, phyloP20way mammalian [20], phastCons7way vertebrate, phastCons 20 way mammalian [21], SiPhy 29way logOdds [22]. However in the real world the situation is not simple. A continuum is observed, ranging from very sever to mild cases. The border between “disease mutation” and “non disease mutation” is artificial and dichotomizing continuous variables is problematic. We decided to address this point and challenge wANNOWAR [3] with a real example, Fabry disease (FD). Mutations that are responsible for this pathology, affect the functioning or the stability of lysosomial alpha-galactosidase (AGAL)(Uniprot: AGAL_HUMAN P06280; EC: 3.2.1.22), which is encoded by the gene *GLA* on the X chromosome.

AGAL is a dimer and its structure has been determined by X-ray crystallography [23–25]. More than 400 missense mutations have been described so far. This number is a surprisingly high value for a protein of 429 aminoacids and almost every amino acid has been found to be mutated. The large genotypic spectrum corresponds to the large phenotypic spectrum of FD, with respect to age at onset, rate of disease progression, severity of clinical manifestations. Patients with the late onset form of FD retain some AGAL activity and are asymptomatic until adult age when they develop cardiac and/or kidney problems [26–29]. When a severe mutation is diagnosed, enzyme replacement must be started even before the symptoms are manifested [30–32], for cases retaining some residual activity, a therapy with small molecules, can be possible [33–35]. Indeed for FD, as well as for other diseases which are due to deficits in lysosomal glycosidases, it is possible to employ iminosugars that stabilize the endogenous protein of the patient acting as pharmacological chaperones or reduce substrate accumulation [36–38]. Iminosugars represent a lucky case of drug repositioning because they were first derived to cure HIV and subsequently used to treat lysosomal storage disorders [24, 39–41].

The classification of FD genotypes is generally carried out on the base of clinical evaluation of patients [42]. Specialized databases such as fabry-database.org [43, 44] annotate mutations with qualitative phenotypes. However a more punctual classification of FD mutations is possible. In fact in order to test the effects of drugs on different mutations, a cell based assay has been developed [45, 46]. Expression vectors encoding mutant AGAL are transiently transfected into COS or HEK293 cells and the residual activity of the enzyme is measured in the extracts of cells that had been treated or not treated with the drug. Residual activity is normalized by the total amount of proteins in the cell (HEK293 or COS) and depends on the stability of the mutant as well as on its specific activity. The ratio between the normalized residual activity of a given mutant and that of wild type AGAL is measured under the same conditions. Part of these data, i.e. those obtained in the absence of the drug, can be “repositioned”, so to speak. They offer the unique possibility of associating a numerical value that correlates to the severity of the damage to hundreds of mutations and consent to evaluate the performance of the most popular predictors of deleterious variant in a realistic scenario of gradual disease severity.

## Methods

Missense *GLA* mutations with phenotypic annotation derived from clinical observation of patients were obtained from a disease specific database of clinical phenotypes and genotypes, fabry-database.org [43, 44] (dataset 1). The mutations (genomic Reference Sequence and protein Reference sequence) and the phenotypes are reported in the 1st, 10th and last column of Additional file 1, respectively.

Missense *GLA* mutations with residual activity annotation were obtained from Fabry_CEP [47, 48]. Relative residual activity is the ratio between the activity measured in cell extracts for a given mutant and the activity of wild type AGAL tranfected into suitable eukaryotic vectors × 100. When residual activity for a given mutation had been measured by more than one lab, the average value was considered (dataset 2). The mutations (genomic Reference Sequence and protein Reference sequence) and the residual activities are reported in the 1st, 10th and last column of Additional file 2, respectively.

The nucleotide numbering on coding DNA Reference Sequence was obtained for each mutation from the appropriate reference link in fabry-database.org or FABRY_CEP.

Nucleotide mutations were mapped onto the reference genome Ensembl GRCh37 release 91 [49]**.**

We performed statistical analysis and data visualisation using the R environment for statistical computing [50].

We calculated descriptive statistics and drew box-and-whiskers plots of residual activity for severe and mild mutations subpopulations using the graphics::boxplot() function on the intersection of the two datasets.

We manually created a confusion matrix using data from the first dataset (175 mutations, from Fabry-database.org, Additional file 1), and measured the goodness of wANNOVAR qualitative predictors using the following indexes:

Raw accuracy: $$ \frac{TP+ TN}{P+N} $$

Balanced Accuracy: $$ 0.5\ \left(\frac{TP}{P}+\frac{TN}{N}\right) $$

F1 score: $$ \frac{2\  TP}{2\  TP+ FP+ FN} $$

Matthew’s correlation coefficient: $\frac{TP\  TN- FP\  FN}{\sqrt{\left( TP+ FP\right)\left( TP+ FN\right)\left( TN+ FP\right)\left( TN+ FN\right)}}$

We coded an R function for the simultaneous calculation of these indexes.

Using the second dataset (280 mutations, manually built), we expressed the correlation between the rank score of tools and residual activity as Pearson’s *r*, and then tested for no correlation using the stats::cor.test() function with ‘less’ alternative (i.e. negative correlation) and the ‘pearson’ method. We drew box-and-whiskers plots of residual activity for every wANNOVAR prediction and conservation tool using the graphics::boxplot() function on the second dataset (280 mutations, manually built).

On the same dataset, we used the graphics::barplot() function for drawing the rank scores of the mutations whose activity is equal or higher than that of wild-type for every wANNOVAR prediction and conservation tool.

## Results

In some cases manifestations of FD occur at an early age with general, neurological, cardiovascular and renal signs, in other cases in adulthood and with a limited subset of symptoms. For this reason a qualitative phenotypic classification of mutations based on the symptoms observed in the patients, has been attempted and classic or severe ones have been distinguished from mild, late onset or variant forms [42].

Fabry-database.org [43, 44] provides a list of mutations and their qualitative phenotypic classification. Since FD is X linked and the association between genotype and phenotype is clearer in males [51], only the 175 hemizygous cases have been gathered from Fabry-database.org and form the first dataset analysed in this paper. The variants were annotated with wANNOVAR [3] and the output is provided in Additional file 1 with the original qualitative phenotypic description in the last column. In the first place it can be noticed that only 51 cases are also present in ClinVar, which is a public archive of reports of the relationships among human variations and phenotypes [52].

To test whether it is possible to broadly distinguish FD mutations collected from Fabry-database.org by the qualitative predictions provided by wANNOVAR annotation, the observed phenotypes were reduced to two classes, a severe group POS of 152 cases, which clusters mutations originally defined as “severe” or “classic”, and a mild group NEG of 23 cases, which clusters those mutations originally defined as “mild”, “late onset”, “variant” or “atypical variant” in fabry-database.org. For the predicted phenotypes, if the tool provides binary classification, like in the case of SIFT [4], the more deleterious one, D in the case of SIFT, is considered as predicted POS, the other one, T in the case of SIFT is considered as predicted NEG. If the tool provides multiple classes, as in the case of PolyPhen-2 [12], the most deleterious one, D in the case of PolyPhen-2, is considered as predicted POS, the other ones, P and B in the case of PolyPhen-2, is considered as predicted NEG. The results are summarized in Table 1. Since the two classes have different sizes, Matthews correlation coefficient should be preferred for the evaluation of predictors [53].

**Table 1:** Accuracy Indexes

Category	Predictor	Raw Accuracy	Balanced accuracy	*F1-score*	Matthew’s correlation coefficient
B	SIFT	0.749	0.549	0.241	0.092
B	LRT	0.794	0.576	0.280	0.162
B	MutationAssessor	0.191	0.460	0.247	−0.106
B	FATHMM	0.846	0.500	0.000	0.000
B	PROVEAN	0.737	0.557	0.258	0.103
Meta	MetaSVM	0.846	0.500	0.000	0.000
Meta	MetaLR	0.846	0.500	0.000	0.000
Meta	M-CAP	0.846	0.500	0.000	0.000
ML	Polyphen2_HDIV	0.771	0.592	0.310	0.175
ML	Polyphen2_HVAR	0.691	0.621	0.341	0.188
ML	MutationTaster	0.194	0.433	0.230	−0.156
ML	FATHMM-MKL	0.829	0.505	0.063	0.022

Accuracy indexes measuring the ability to differentiate severe from mild *GLA* mutations for all the predictors used by wANNOVAR. Categories are B for “biologically based prediction method”, ML for “Machine Learning based prediction method”, and Meta for “Meta prediction method”

For most tools the values are quite low and in some cases no discrimination is possible.

A different way of ordering by severity, relies on the residual activity of AGAL mutants measured in vitro in HEK293 or COS cell transiently transfected with expression plasmids. Values for 280 mutations have been collected gathering results of several laboratories [33, 45, 54–62]. They form the second dataset analyzed in this paper. wANNOVAR annotation for these mutants can be found in Additional file 2 with the relative residual activity in the last column.

**Fig. 1:**
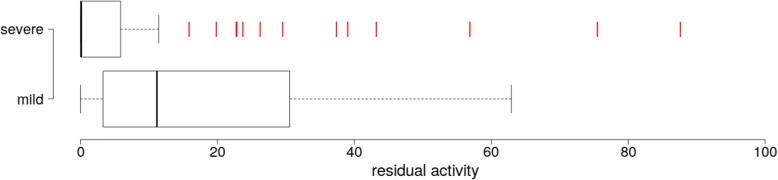
Distribution of residual activities for phenotypically annotated *GLA* mutations. The boxplot shows the distribution of residual activity in the subpopulations of mutations with severe and mild effects. The red bars represent outliers

The intersection between the two datasets is formed by 67 mutations of the severe group POS and 12 of the mild group NEG, for which relative residual activity is available. The median residual activity of severe mutations POS is 0.1 (Fig. 1). This finding suggests that severe cases have null, or very close to null activity, when tested in transfected cells. The box plot in Fig. 1 shows 20% outliers with high residual activity in POS population that might represent an overestimation in the original literature.

Contrary to what occurs in the first dataset of mutations whose phenotypic annotation is derived from clinical literature (Additional file 1), the second dataset, whose annotation is based on residual activity (Additional file 2), is balanced with half of the mutations with values above 0.

**Fig. 2:**
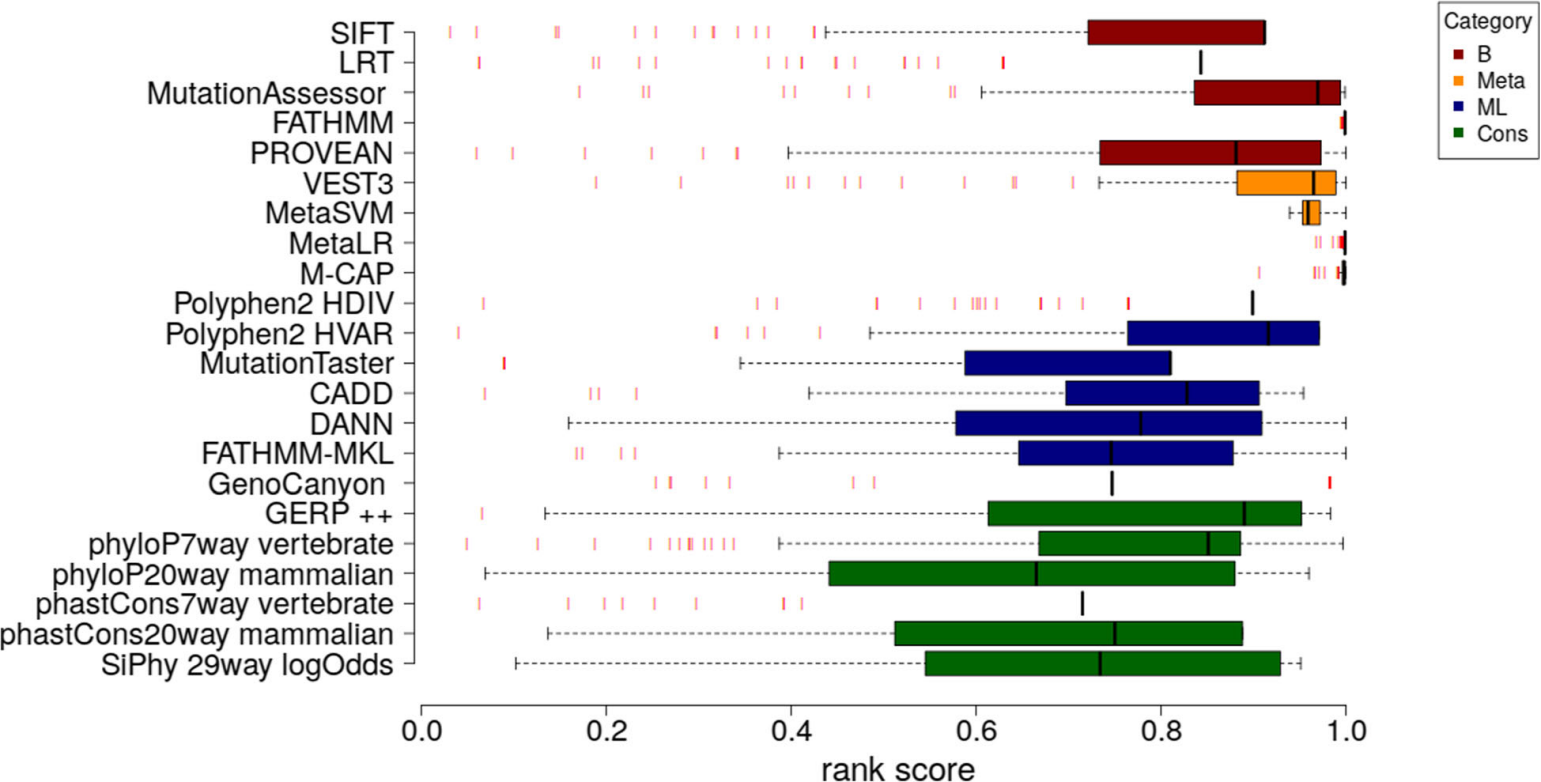
Distribution of rank scores for mutations with null residual activity. The boxplot show the distribution of the rank scores for all the predictors used by wANNOVAR. The red bars represent outliers. Predictor category label is B for “biologically based prediction method”, ML for “Machine Learning based prediction method”, Meta for “Meta prediction method” and Cons for “Conservation scoring tool”

**Fig. 3:**
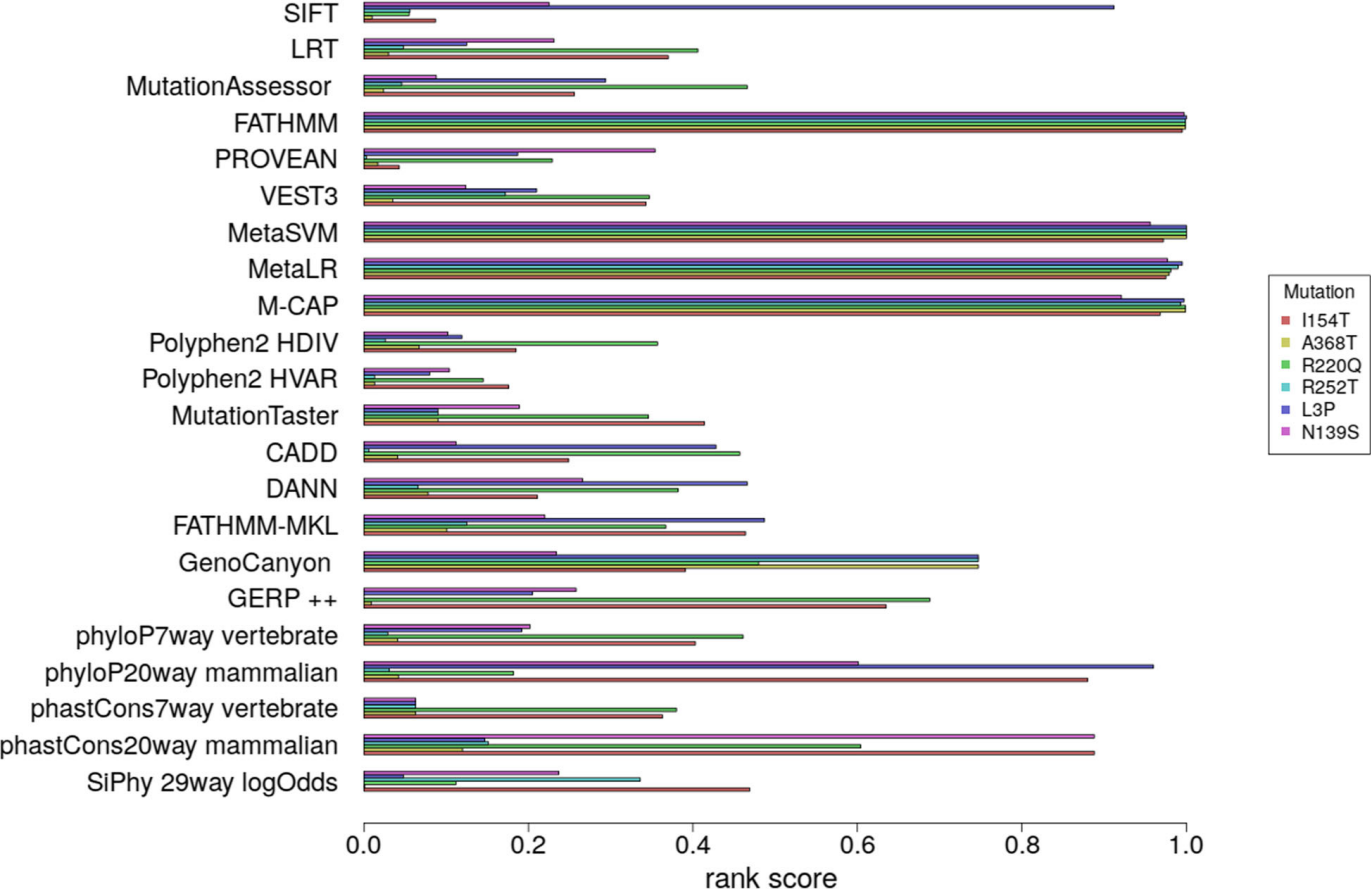
Rank scores for mutations with residual activity equal or greater than wild type alpha-galactosidase. The histograms show the rank scores of the six mutations whose residual activity is greater or equal than the wild type alpha-galactosidase, for each of the wANNOVAR predictors. Mutations are color coded, and are detailed inset

The box plot in Fig. 2 shows the distribution of rank scores for mutations showing 0 residual activity. Rank scores were created by wANNOVAR to make the functional prediction scores and conservation scores more comparable to each other and monotonic (a higher score indicating “more likely to be damaging”) [63]. As can be observed FATHMM [7], metaSVM [10], metaLR [10], M-CAP [11]correctly assign high scores to very severe cases. On the other side, the histograms in Fig. 3 show the rank scores assigned by the predictors to 6 non pathological mutation whose residual activity is comparable or higher than that of wild type. The same predictors, FATHMM, metaSVM [10], metaLR [10], M-CAP [11], give a constantly high score and tend to over-estimate the damage caused by a mutation. In Table 2 the correlation between the rank scores of the predicting tools and the residual activity of all the mutations in the second dataset (Additional file 2), is shown. Results obtained by some predictors used by wANNOVAR, for example VEST3 (Pearson correlation coefficient 0.71; *p* < 0.0001) and PolyPhen-2 (Pearson correlation coefficient − 0.62; *p* < 0.0001), demonstrate that the rank scores can correlate with severity in a statistically significant manner. Methods based on evolutionary and phylogenetic analysis perform very poorly.

**Table 2:** Correlations

Category	Name	Pearson’s *r*	*p-value*
B	SIFT	− 0.493	7.87E-19
B	LRT	−0.486	2.76E-18
B	MutationAssessor	−0.573	5.22E-26
B	FATHMM	−0.054	1.85E-01
B	PROVEAN	−0.546	1.86E-23
Meta	VEST3	**−0.699**	1.08E-42
Meta	MetaSVM	0.285	1.00E + 00
Meta	MetaLR	−0.482	5.77E-18
Meta	M-CAP	−0.255	8.09E-06
ML	POLYPHEN2 HDIV	**−0.672**	1.67E-38
ML	POLYPHEN2 HVAR	**−0.648**	4.53E-35
ML	MutationTaster	−0.499	2.42E-19
ML	CADD	−0.595	1.78E-28
ML	DANN	−0.388	8.51E-12
ML	FATHMM-MKL	−0.434	1.35E-14
ML	GenoCanyon	−0.282	7.95E-07
n	GERP++	−0.405	9.34E-13
Cons	phyloP7way vertebrate	−0.441	4.79E-15
Cons	phyloP20way mammalian	−0.214	1.54E-04
Cons	phastCons7way vertebrate	−0.486	2.55E-18
Cons	phastCons 20 way mammalian	−0.256	7.35E-06
Cons	SiPhy 29way logOdds	−0.389	7.65E-12

Pearson’s *r* correlation coefficient between rank scores and residual activities, together with the associated *p*-value for significance scoring, for all the predictors used by wANNOVAR. Bold text is used for the highest correlations. Categories are B for “biologically based prediction method”, ML for “Machine Learning based prediction method”, Meta for “Meta prediction method” and Cons for “Conservation scoring tool”

## Discussion

The gene *GLA* offers an example of the critical points encountered when missense mutations are annotated. In the first place getting the phenotype associated to a mutation is difficult, most information is still missing in databases such as ClinVar [52] and is present only in specialized databases. Mild and severe mutations can be mis-classified in the literature. An example is provided by the mutation D313Y, that is reported as “classic” in fabry-database.org, but is regarded as “likely benign/uncertain significance” in ClinVar [52] and is relatively frequent in the population according to ExAC [64] and 1000Genomes [65] (Additional file 1). The residual activity of D313Y is as high as 75% than wild type (Additional file 2) thus suggesting that the interpretation of fabry-database.org, which is derived from the original source [66], is overestimated. Other examples are provided by the outliers in Fig. 1. Given these premises, it is not surprising that the tools provided by wANNOWAR cannot distinguish mild from severe mutations as they are defined in the literature.

Hence to train or test algorithms that can grade disease severity, datasets of quantitative measures of the damage caused by mutations to the proteins must be available. In this paper we used data produced by a cell based assay that measures relative residual activity in the cells. We showed that some of the popular tools used for exome analysis, are able to grade disease severity, even though they had not been trained or tested for this specific purpose. A summary of all the tools employed in this study is provided in Additional file 3. The best result was obtained with VEST3 [9] that uses a supervised machine learning algorithm, Random Forest based on 86 sequence features and trained with a positive class of missense variants from the Human Gene Mutation Database and a negative class of common missense variants detected in the Exome Sequencing Project population. In a recent paper Plon and co-workers [67] compared the performance of several algorithms using benign or pathogenic missense variants from the ClinVar database [52]. They found “poor concordance among algorithms, particularly for variants classified as benign by clinical laboratories”. Nevertheless they observed that VEST3 has the lowest rate of false positive calls, i.e. benign variants in ClinVar that are erroneously predicted as pathogenic. This finding suggests that the training protocol employed by VEST3 reduces over-prediction of deleterious variants. The second best result was obtained with PolyPhen-2 [12] that calculates bayesian probabilities and uses eight sequence-based and three structure-based predictive features. Since AGAL structure is known [23–25], it is possible that the incorporation of structure-based predictive features contributed to the good results obtained with PolyPhen-2. Two versions of the same program exist. PolyPhen-2 HumDiv is trained with a positive class of mutation causing Mendelian diseases from UniProt and a negative class of variants found in closely related mammalian homologs whereas PolyPhen-2 HumVar is trained with a positive class consisting of all human disease-causing mutations from UniProt and a negative class consisting of nsSNPs without annotated involvement in disease. HumDiv performed slightly better than HumVar. Among the tools that are not limited to exonic missense mutations, CADD is the best performing one. MutationAssessor is the best performing method based on biological principles with a combinatorial entropy formalism. In a previous paper we had shown that the flexibility of the residue where the mutation occurs is the best structural property to predict AGAL mutants residual activity [68]. Results obtained by VEST3, PolyPhen-2, CADD and MutationAssessor are better than those obtained with molecular dynamics (Pearson correlation coefficient R 0.50; *p* < 0.0001). Although the majority of disease mutations in *GLA* affect protein stability, methods based on a single structural property perform worse than those relying on several properties.

Admittedly our analysis has two major limitations. In the first place only the programs run by wANNOVAR [3] were considered leaving out those softwares that use three-dimensional structures, for example SDM [69], PoPMuSiC [70] and mCSM [71]. In the second place only one gene was considered. Yet *GLA* represents a unique case since, to the best of our knowledge, there are few data about residual activity of other mutant proteins. We hope that the effort that was put in place for *GLA* were extended.

## Conclusions

Our paper aims at soliciting a combined effort to produce a large database where the residual activity measured in a cell-based test for diverse proteins is gathered. Indeed this is feasible if cDNA encoding mutants are expressed by transient transfection in suitable mammalian cells. In case of FD, it has been shown that this in vitro test recapitulates what can be observed ex vivo in the cells derived from patients. This approach is not limited to the variants already observed in the patients or in the healthy population and provides data for negative controls too, i.e. mutation that do not affect residual activity. One obvious limitation of the method is that the effect of exonic mutation affecting splicing cannot be evaluated. Once a large dataset from diverse genes is gathered, it could be used to train linear classifiers. We also suggests that programs relying on several features, including structure-based ones, are included in the tools used for the high throughput annotation of data deriving from exome sequencing.

## Additional files

Additional file 1: wANNOVAR annotated GLA mutation with qualitative phenotypes. (XLSX 121 kb)

Additional file 2: wANNOVAR annotated GLA mutation with relative residual activities. (XLSX 212 kb)

Additional file 3 Summary of the predictors evaluated in this study and their main characteristics (XLSX 13 kb)

## Abbreviations

AGAL: lysosomial alpha-galactosidase
FD: Fabry disease
